# Heterogeneity in form and function of the rat extensor digitorum longus motor unit

**DOI:** 10.1111/joa.13590

**Published:** 2021-11-10

**Authors:** Roger W. P. Kissane, Samit Chakrabarty, Graham N. Askew, Stuart Egginton

**Affiliations:** ^1^ Department of Musculoskeletal & Ageing Science University of Liverpool Liverpool UK; ^2^ School of Biomedical Sciences University of Leeds Leeds UK

**Keywords:** capillary supply, motoneuron, oxygen modelling, skeletal muscle, work loop

## Abstract

The motor unit comprises a variable number of muscle fibres that connect through myelinated nerve fibres to a motoneuron (MN), the central drivers of activity. At the simplest level of organisation there exist phenotypically distinct MNs that activate corresponding muscle fibre types, but within an individual motor pool there typically exists a mixed population of fast and slow firing MNs, innervating groups of Type II and Type I fibres, respectively. Characterising the heterogeneity across multiple levels of motor unit organisation is critical to understanding changes that occur in response to physiological and pathological perturbations. Through a comprehensive assessment of muscle histology and ex vivo function, mathematical modelling and neuronal tracing, we demonstrate regional heterogeneities at the level of the MN, muscle fibre type composition and oxygen delivery kinetics of the rat extensor digitorum longus (EDL) muscle. Specifically, the EDL contains two phenotypically distinct regions: a relatively oxidative medial and a more glycolytic lateral compartment. Smaller muscle fibres in the medial compartment, in combination with a greater local capillary density, preserve tissue O_2_ partial pressure (PO_2_) during modelled activity. Conversely, capillary supply to the lateral compartment is calculated to be insufficient to defend active muscle PO_2_ but is likely optimised to facilitate metabolite removal. Simulation of in vivo muscle length change and phasic activation suggest that both compartments are able to generate similar net power. However, retrograde tracing demonstrates (counter to previous observations) that a negative relationship between soma size and C‐bouton density exists. Finally, we confirm a lack of specificity of SK3 expression to slow MNs. Together, these data provide a reference for heterogeneities across the rat EDL motor unit and re‐emphasise the importance of sampling technique.

## Graphical abstract

Labelled motoneurons (MNs) across the extensor digitorum longus (EDL) motor pool highlight the negative relationship between soma area and vesicular acetylcholine transporter Density and SK3 Density. These MNs innervate phenotypically distinct portions of the EDL muscle that is synchronously recruited to provide power for movement. At the level of individual fibres and capillaries heterogeneities in oxygen supply and demand are evident, which when scaled up combine to meet the demands of the muscle, principally maintenance of oxidative phosphorylation in the medial and removal of lactate in the lateral compartment.

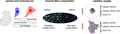

## 1 INTRODUCTION

Physiology is inherently variable, e.g. anatomical heterogeneities of endothelial cells across specific vascular beds (Vanlandewijck et al., 2018), sites of reactive oxygen species production within skeletal muscle (Staunton et al., 2019) and distribution of calcium exchange channels within cardiomyocytes (Jayasinghe et al., 2009). Understanding the origin of such heterogeneities within an experimental system is essential to be able to accurately assess the integrated response to physiological or pathological perturbations.

A commonly utilised skeletal muscle in experimental physiology and biomedical research is the rat extensor digitorum longus (EDL) muscle, utilised in studies of e.g. ischaemia (Tickle et al., 2020), heart failure (Espino‐Gonzalez et al., 2021), ageing (Brown et al., 1992) and biomechanics (Eddinger et al., 1985; Kissane et al., 2018; Luff, 1981). The EDL is active during the swing phase of walking and thought to be involved in dorsiflexion of the ankle (Nicolopoulos‐Stournaras & Iles, 1984). This fusiform muscle comprises a single proximal tendon, with the distal end subdividing into four separate muscle‐tendon compartments, with tendons that insert onto the phalanges of digits II–V. These individual compartments represent different proportions of muscle mass, with heads II–V connected to 23%, 17%; 15% and 45% of the EDL muscle mass, respectively (Huijing et al., 1998). Interestingly, this highly compartmentalised mixed hindlimb muscle is often treated as a relatively homogeneous entity. However, a brief report (Egginton, 1990) on phenotypic variation in the rat EDL described regional heterogeneity where, depending on the sampling indices adopted, different conclusions may be drawn about muscle composition.

Little progress has been made to better characterise EDL structural and functional composition in the last three decades. We confirmed regional heterogeneity in fibre type composition of the EDL (Deveci et al., 2001; Kissane & Egginton, 2019), linked with regional‐specific adaptations to systemic hypoxia (Deveci et al., 2001), and demonstrated distinct optimal frequency to generate peak power during cyclical contractions within EDL compartments associated with different responses to fatigue (Kissane et al., 2018). However, there lacks a comprehensive description of the local capillary supply across this supposedly heterogeneous muscle. Additionally, there remains a paucity of information regarding distribution of the central drivers of activity (i.e. the motoneuron; MN) that determine the phenotypic arrangement of muscle. Understanding fine scale heterogeneities in structure and function among the whole motor unit is needed to meaningfully quantify maladaptive changes associated with pathologies like ageing and motor neuron disease, where Type IIb/x units are especially susceptible to denervation and dysfunction (Hepple & Rice, 2016; Kanning et al., 2010).

Here we quantified the structural composition of the most medial (head II) and lateral (head V) compartments of the rat EDL using histological assessment of muscle phenotype and local vascular supply, with functional consequence inferred from mathematical modelling of oxygen consumption. Using the muscle work loop technique to replicate in vivo cyclical muscle length change trajectories and phasic activation, we provide an assessment of in situ capacity to generate power. Finally, compartmentally injected retrograde tracers in combination with histological assessment of C‐bouton complex SK3 (Deardorff et al., 2013, 2014; Smith & Brownstone, 2020) was used to characterise phenotypically distinct MN populations.

## 2 METHODS

### 2.1 Animals

Male Wistar rats (6–8 weeks old) were used in this study, housed under a 12:12 light–dark cycle in a temperature‐controlled 21°C environment, with ad libitum access to food and water. Six animals were used to characterise the isolated muscle mechanical properties (226 ± 11 g) with seven (261 ± 8 g) used for MN tracing and histological assessment of muscle fibre type and vascular composition.

### 2.2 Isolated muscle experiments

Briefly, animals were anaesthetised with isoflurane (5% in 100% O_2;_ IsoFlo^®^; Zoetis UK Ltd) and maintained at 2% during the careful dissection of the hindlimb EDL muscle. The distal tendons that insert onto the phalanges of digits II–V (Figure S1a) were located, released by transecting the retinaculum ligament in the ankle, and the tibialis anterior muscle removed to expose the EDL (Figure S1b). The isolated EDL was placed immediately into chilled (4°C), oxygenated (95% O_2_, 5% CO_2_) Krebs–Henseleit solution (in mmol L^−1^: 117 NaCl, 4.7 KCl, 2.5 CaCl_2_, 1.2 MgSO_4_, 24.8 NaHCO_3_, 1.2 KH_2_PO_4_ and 11.1 glucose) (Burton, 1975), and the animal culled by an approved schedule one technique. The whole EDL was pinned out on a Sylgard^®^ (761036; Sigma) lined Petri dish at approximately in vivo resting length (Figure S1c). The most medial (21.1 ± 0.79 mg) and lateral (51.1 ± 9.5 mg) compartments of the muscle were dissected free under a dissection microscope following fascicles that insert onto each distal tendon towards the proximal tendon. These remained in oxygenated Krebs–Henseleit solution until experimentation (Figure S1d) while the two intermediate compartments were discarded. At random, muscle compartments were attached to a metal rod fixed to a polychlorotrifluoroethylene lid and placed into a glass chamber containing fresh oxygenated Krebs–Henseleit solution. The muscle was then connected *via* a light stainless steel rod to the arm of an ergometer (305B‐LR; Aurora Scientific Inc.) mounted to a Digimatic height gauge (Mitutoyo UK, Ltd) to control muscle length, and allowed to recover for 30 min before the experiments.

### 2.3 Isometric muscle mechanics

Muscle length was incrementally increased in 0.5 mm steps to determine the twitch force–length relationship and therefore identify the length at which maximum isometric twitch force was generated (*L*_0_). The muscle was activated using a supramaximal stimulus with a 0.2 ms pulse width using parallel platinum electrodes. A 200 ms isometric tetanus (200 Hz stimulus frequency) was performed and used to calculate maximum isometric tetanic stress. This length was used in the subsequent work loop experiment.

### 2.4 Mechanical power during cyclical contractions

Using the work loop technique (Josephson, 1985), muscles were subjected to cyclical length changes and phasic electrical stimulation to activate muscles such that force generation occurred predominantly during shortening, maximising the net work and power generated by the muscle. The functional capacity of individual muscle compartments to generate power during five sinusoidal length trajectory at 7 Hz was compared with mean length *L*_0_ and a strain amplitude of ±5% fibre length, as previously reported (Kissane et al., 2018). Net power was calculated from the average of the two greatest cycles across the five work loop cycles.

### 2.5 MN tracing

Animal surgery was completed by a competent Home Office approved PIL holder, under aseptic conditions. Surgical anaesthesia was induced and maintained with isoflurane (5% and 2%, respectively, in 100% O_2_; IsoFlo; Zoetis UK Ltd). Two retrograde fluorescent tracers were injected into the right EDL 5 days prior to muscle sampling. The medial injections are positioned more proximally in the EDL muscle compared with the lateral (see Figure S1) with the medial compartments deep tendon/aponeurosis used to direct the needle for tracers, compared with the lateral injections which are more distally placed. A volume of 1 μl of 1.5% 647 nm cholera toxin subunit b (CTB) Alexa Fluor™ Conjugate (Invitrogen) was injected into both medial and lateral compartments and 3 μl of 1.5% 555 nm Fast Blue (FB; Polyscience, Inc.) was injected only into the medial EDL compartment. The combinatorial tracer approach allows us to differentially identify MNs from the lateral compartment (CTB^+^.FB^−^) and medial compartment (CTB^+^.FB^+^). The skin was sutured using 5‐0 Mersilk (Ethicon; Johnson & Johnson Medical Ltd). Animals received analgesic (0.015 mg/kg, Vetagesic^®^; Ceva) and antibiotic (2.5 mg/kg, Baytril^®^; Baye) subcutaneously for 2 days post‐surgery.

### 2.6 Tissue preparation

Animals were anaesthetised and the right EDL was dissected, the mid‐portion was coated in optimum cutting temperature compound (OCT, Agar Scientific) then snap frozen in isopentane cooled in liquid nitrogen. All muscle tissue was stored at −80°C until cryo‐sectioning. Next, animals were transcardially perfused with 0.1 M phosphate buffer and fixed with 4% paraformaldehyde (PFA). Spinal columns were removed immediately after perfusion and post‐fixed in 4% PFA for 24 h. Spinal cords were dissected and cryoprotected in 30% sucrose at 4°C for 7 days. Next, lumbar segments were isolated, frozen in OCT (Agar Scientific) and stored at −20°C.

### 2.7 Muscle histology

Extensor digitorum longus muscles were cryo‐sectioned (−20°C, 12 µm), mounted on polylysine‐coated slides (VWR International) and stored at −20°C until staining. Muscle fibre type composition was determined using methods previously described (Al‐Shammari et al., 2019; Kissane et al., 2018). Briefly, the basement membrane was labelled with an anti‐laminin antibody (Sigma‐Aldrich; L9393) to identify fibre boundaries, while monoclonal myosin heavy chain antibodies were used to simultaneously label Type I (BAD5; Developmental Studies Hybridoma Bank, University of Iowa) and Type IIa (SC‐71; Developmental Studies Hybridoma Bank, University of Iowa) fibres. Unstained fibres were categorised as Type IIb/x. Capillaries were labelled by fluorescein‐conjugated *Griffonia simplicifolia* lectin I (Vector Laboratories; FL‐1101), an endothelial cell carbohydrate‐binding protein. Photomicrographs were taken with a QImaging MicroPublisher 5.0 RTV camera (Teledyne QImaging) on a Nikon Eclipse E600 microscope (Nikon) at 20× magnification (440 × 330 μm field of view). Two regions of interest were taken at the extreme lateral and medial edges of the EDL to calculate compartmental indices of fibre type composition and vascular supply (Kissane et al., 2018).

Indices for capillary‐to‐fibre ratio (C:F) and capillary density (CD) and fibre cross‐sectional area (FCSA) were derived from histological sections. The global indices presented here are most common among basic and pathological studies describing the relationship between capillaries and muscle fibres; C:F may provide information relating to capillary growth/rarefaction, while CD may provide information on the diffusive capacity of the capillary bed. However, while these global indices describe gross changes in capillary supply they lack descriptive power for capillary distribution, which has a significant impact on the functional capacity of muscle (Al‐Shammari et al., 2019; Kissane et al., 2021). Therefore, we present data describing the local capillary supply (assessed as capillary domain area, CDA; local capillary to fibre ratio, LCFR; or local capillary density, LCD) as a critical determinant of functional capacity (Al‐Shammari et al., 2019; Kissane et al., 2021). To investigate the functional consequence of heterogeneous capillary supply and oxygen demand we mathematically modelled skeletal muscle oxygen transport kinetics using the publicly available oxygen transport modeller (OTM; Al‐Shammari et al., 2019). Briefly, using fibre boundary (laminin‐labelled tissue) and capillary locations (lectin positive microvessels), the OTM generates a digital mask of the skeletal muscle cross section, which incorporated individual fibre types to provide fibre type‐specific oxygen demand (Sullivan & Pittman, 1984; Wüst et al., 2009) allowing us to model local oxygen consumption and estimate tissue O_2_ partial pressure (PO_2_) distribution.

### 2.8 Spinal cord immunohistochemistry

Spinal cord immunohistochemistry was performed as previously described (Kissane, Al‐Shammari, et al., 2021; Smith et al., 2017). In brief, the L3–L6 segments were sectioned at 50 µm on a cryostat, and free‐floating sections were collected and stored in phosphate‐buffered saline (PBS) until staining. They were then washed in PBS (3 x 10 min), and incubated for 1‐h in blocking solution (0.2% Triton X‐100, PBS, NaCl and 10% normal donkey serum). The free‐floating sections were then incubated for 48 h in primary antibodies diluted in blocking solution, washed and then incubated in secondary antibodies for 2 h, also in blocking solution. Primary antibodies: goat anti‐vesicular acetylcholine transporter (anti‐VAChT; Millipore; Cat# ABN100, RRID:AB_2630394, 1:1000) to identify C‐bouton synapses and rabbit anti‐SK3 (Millipore; Cat# AB5350‐200UL, RRID:AB_91797, 1:200) to identify potassium channel SK3. Secondary antibodies at 1:200: Alexa Fluor^®^ 488 donkey anti‐goat (Jackson ImmunoResearch Labs; Cat# 705–546–147, RRID:AB_2340430) and Alexa Fluor 555 donkey anti‐rabbit 555nm (AB_2563181) were used to label VAChT and SK3, respectively. Finally, tissue was mounted on glass slides with Mowiol 4‐88 (Carl Roth GmbH & Co. Kg).

### 2.9 Confocal microscopy and quantitative analysis of the spinal cord

Images were acquired with a Zeiss LSM 800 confocal microscope (Zeiss LSM 800 with Airyscan, RRID:SCR_015963), with a 40× oil immersion objective (1 AU aperture), and Zeiss ZEN Blue Edition software (ZEN Digital Imaging for Light Microscopy, RRID:SCR_013672). MNs were identified by their location in the spinal cord ventral horn and presence of CTB (647 nm) or FB (405 nm) staining. Z‐stacks of 30 μm at 0.40 μm intervals were acquired through the centre of each neuron, identified by the nucleus.

Three‐dimensional (3D) reconstructions of each MN were rendered from the confocal image Z‐stacks, utilising Imaris Software. In the 3D isometric view, solid surfaces of the MN soma with dendrites, C‐boutons and SK3 were created *via* surface rendering and thresholding. CTB or FB was used to model the MN surface. A masking feature was then used to select SK3 clusters contacting the MN surface and/or proximal to the C‐bouton. Imaris was then used to generate volume and surface area data for each MN, C‐bouton and SK3 cluster. To determine the MN cross‐sectional area, the soma perimeter was outlined using ImageJ at the central plane of the nucleus, as illustrated elsewhere (Kissane et al., 2021).

Since all alpha‐MNs contain C‐boutons, cells with no C‐bouton labelling were removed (Deardorff et al., 2014). To identify medial *vs*. lateral MNs, a fluorescence signal intensity threshold was measured in IMARIS. Cells with a fast blue intensity greater than 2785 intensity units (AU) were classified as FB‐positive, and therefore medial EDL MNs. All other CTB^+^, FB^−^ MNs were classed as lateral MNs. The threshold used for segregating MN was chosen by finding the greatest drop off in intensity using histograms created using the following equation:

$$\left[\text{(maximum}‐\text{minimum FB)}/\left(\sqrt{\text{number of motorneurons}}\right)\right]$$

All data are expressed as mean ± standard error. Statistical test (Shapiro‐Wilks) for normality was completed on all data, where either an independent *t*‐test (normally distributed) or Mann–Whitney U tests (not normally distributed) were completed. Relationships between local capillary indices and fibre size, as well as synapse density and soma area were assessed using linear regressions. All statistical analysis was performed using IBM SPSS Statistics, v.25, with statistical significance set at *p* < 0.05.

## 3 RESULTS

### 3.1 Phenotypically distinct compartments of the EDL

**FIGURE 1:**
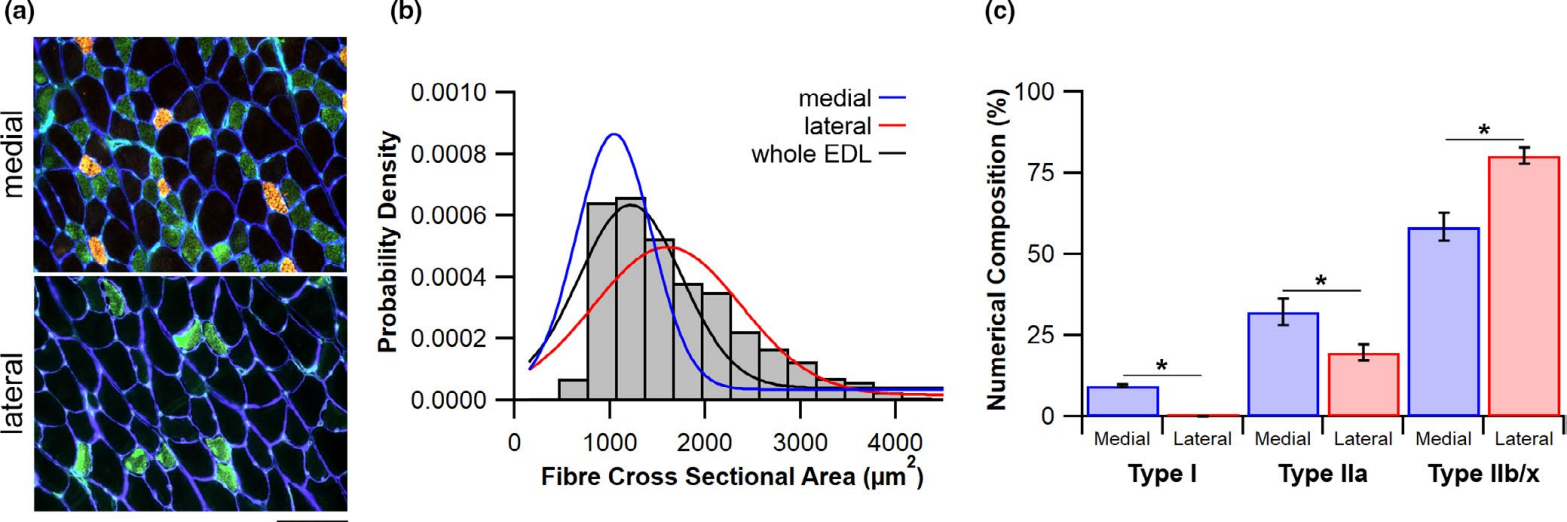
Phenotypical arrangement of the extensor digitorum longus (EDL) muscle. (a) Histological micrographs taken in the medial and lateral compartments showing Type I (red), Type IIa (green) and Type IIb/x (unstained) fibres; scale bar = 100 µm. (b) Probability density distribution of fibre cross‐sectional area across the EDL with Gaussian plots for the whole muscle (black), the medial fibres (blue) and the lateral fibres (red). (c) Numerical composition of fibres contained within the different portions of the EDL. Mean ± standard error of the mean (SEM), *p < 0.05

The EDL has a distinctly heterogeneous fibre type distribution, graded from the oxidative medial compartment laterally to the more glycolytic portion of the muscle (Figure 1a). Fibre size varies according to location (Figure 1b) with the medial compartment average fibre area 39% smaller than that of the lateral [1308 ± 65 vs. 1824 ± 145 µm^2^, respectively; *t*(10) = −3.251, *p* = 0.009]. In addition, there exists a significant phenotypic difference in fibre type composition between medial and lateral compartments (Figure 1c; Table 1). The lateral compartment comprises entirely of Type II fibres, with a numerical composition of 19.8 ± 2.5% Type IIa and 80.2 ± 2.5% Type IIb/x compared with the more oxidative medial compartment containing 9.4 ± 0.5% Type I, 32.3 ± 4.2% Type IIa and 58.4 ± 4.3% Type IIb/x. Interestingly, when looking at the areal composition of the muscle fibres, the lateral compartment consists of only 10.5 ± 1.7% Type IIa fibres, with the remaining 89.5 ± 1.7% Type IIb.

**TABLE 1:** Muscle fibre morphometrics for the individual compartments of the EDL

	Medial	Lateral	*t*	*p* value
Type I area (µm^2^)	775 ± 73	0.0 ± 0.0	10.6641	0
Type IIa area (µm^2^)	862 ± 70	953 ± 90	−0.797	0.444
Type IIb/x area (µm^2^)	1702 ± 139	2059 ± 197	−1.482	0.169
Type I area composition (%)	5.50 ± 0.50	0.0 ± 0.0	11.106	0
Type IIa area composition (%)	21.04 ± 3.21	10.45 ± 1.67	2.931	0.020
Type IIb/x area composition (%)	73.46 ± 3.44	89.55 ± 1.67	−4.206	0.004

Note

Values presented are mean ± SEM (*n* = 6/group).

Abbreviations: EDL, extensor digitorum longus; SEM, standard error of the mean.

### 3.2 Heterogeneity in capillary distribution and oxygen tension profile

**FIGURE 2:**
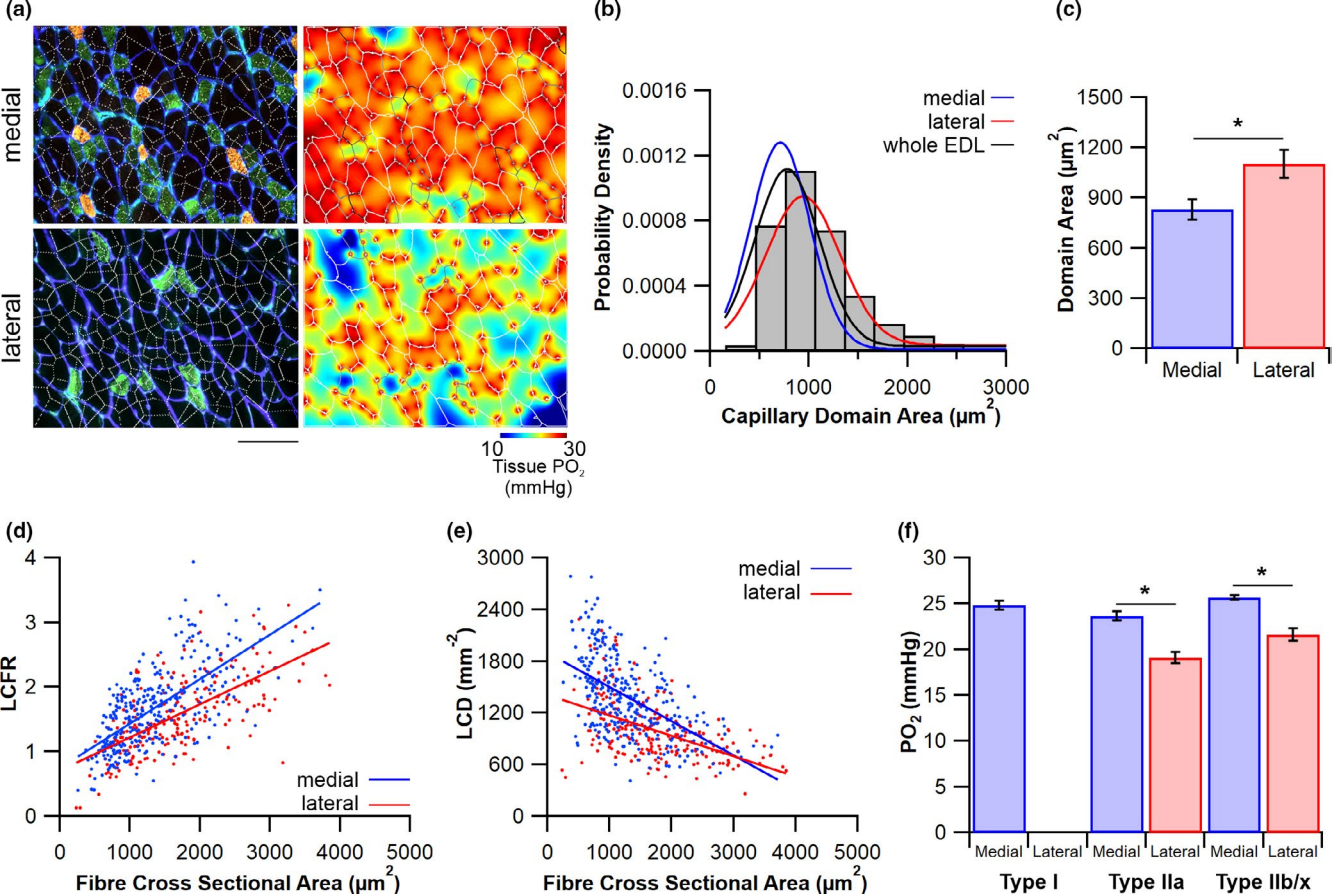
Heterogeneity in capillary supply. (a) Capillary domain areas (white) overlaid onto histological micrographs of the medial and lateral compartment. Using the publicly available oxygen transport modeller (Al‐Shammari et al., 2019) mathematical predictions of tissue PO_2_ are calculated using individual capillary locations as a modelled point source of oxygen, with muscle fibre type‐specific oxygen demands. The figures are the resultant oxygen tension presented as heatmaps of PO_2_ across the muscle fibres (white boundaries); scale bar = 100 µm. Frequency distribution of the capillary domain area across the whole extensor digitorum longus (EDL) (b) with Gaussian plots for the whole EDL (black), the medial fibres (blue) and the lateral fibres (red). Local indices of capillary supply are presented as the average capillary domain area (c), plus the relationship between fibre cross‐sectional area and local capillary to fibre ratio (LCFR) (d) and local capillary density (LCD) (e). Finally, predictions for mean fibre PO_2_ across all three major fibre types within each compartment (f). Mean ± standard error of the mean (SEM), *p < 0.05

Each muscle fibre type has a different level of oxygen demand; therefore, a heterogeneity in muscle fibre type distribution implies a gradient in oxygen demand across the muscle. This presumably requires a tailored microvascular supply. Indeed, the lack of difference in C:F in combination with a significantly elevated CD in the more oxidative medial compartment suggests that the microvascular supply is determined by local fibre morphology and fibre type distribution (Table 2). However, to resolve functional heterogeneities in oxygen supply at an individual fibre level we utilised scale‐independent local indices in combination with mathematical modelling of resulting oxygen tension, using anatomically accurate capillary and fibre spatial distributions (Figure 2a). There was a significantly lower capillary supply (domain) area in the medial (831 ± 61 mm^−2^) compared with the lateral compartment of the EDL [1102 ± 83 mm^−2^, *t*(10) = −2.929, *p* = 0.015, Figure 2b,c].

**TABLE 2:** Indices of capillary supply for the medial and lateral compartments of the EDL

	Medial	Lateral	*t*	*p* value
Global capillary indices				
C:F	1.54 ± 0.07	1.57 ± 0.09	−0.304	0.768
CD (mm^−2^)	1297 ± 62	914 ± 74	3.961	0.003
Local capillary indices				
All fibres LCFR	1.65 ± 0.05	1.64 ± 0.07	0.111	0.914
Type I LCFR	1.22 ± 0.07	0.0 ± 0.0	17.763	0
Type IIa LCFR	1.25 ± 0.04	1.20 ± 0.12	0.403	0.695
Type IIb/x LCFR	1.95 ± 0.10	1.76 ± 0.08	1.576	0.146
All fibres LCD (mm^−2^)	1352 ± 93	974 ± 78	3.107	0.011
Type I LCD (mm^−2^)	1657 ± 123	0.0 ± 0.0	13.451	0
Type IIa LCD (mm^−2^)	1498 ± 95	1282 ± 114	1.46	0.175
Type IIb/x LCD (mm^−2^)	1228 ± 108	907 ± 77	2.418	0.036

Note

Values presented are mean ± SEM (*n* = 6/group).

Abbreviations: CD, capillary density; C:F, capillary to fibre ratio; LCD, local capillary density; LCFR, local capillary to fibre ratio; SEM, standard error of the mean.

There is a clear positive relationship between LCFR and FCSA for both the medial (*R* ^2^ = 0.472, *p* < 0.001) and lateral (*R* ^2^ = 0.44, *p* < 0.001, Figure 2d) compartments, while LCD demonstrates a negative relationship with FCSA across the medial (*R* ^2^ = 0.304, *p* < 0.001) and lateral compartments (*R* ^2^ = 0.249, *p* < 0.001, Figure 2e). Fibre type‐specific local indices of capillary supply are presented in Table 2. The combination of lower CDA and higher LCD (Table 2) is consistent with an improved tissue oxygen profile (Figure 2a,f), with the average fibre PO_2_ higher in the medial compared with the lateral muscle compartment [25.2 ± 0.2 vs. 21.4 ± 0.7 mmHg, *t*(10) = 5.46, *p* < 0.0001].

### 3.3 Mechanical properties of the EDL compartments

**FIGURE 3:**
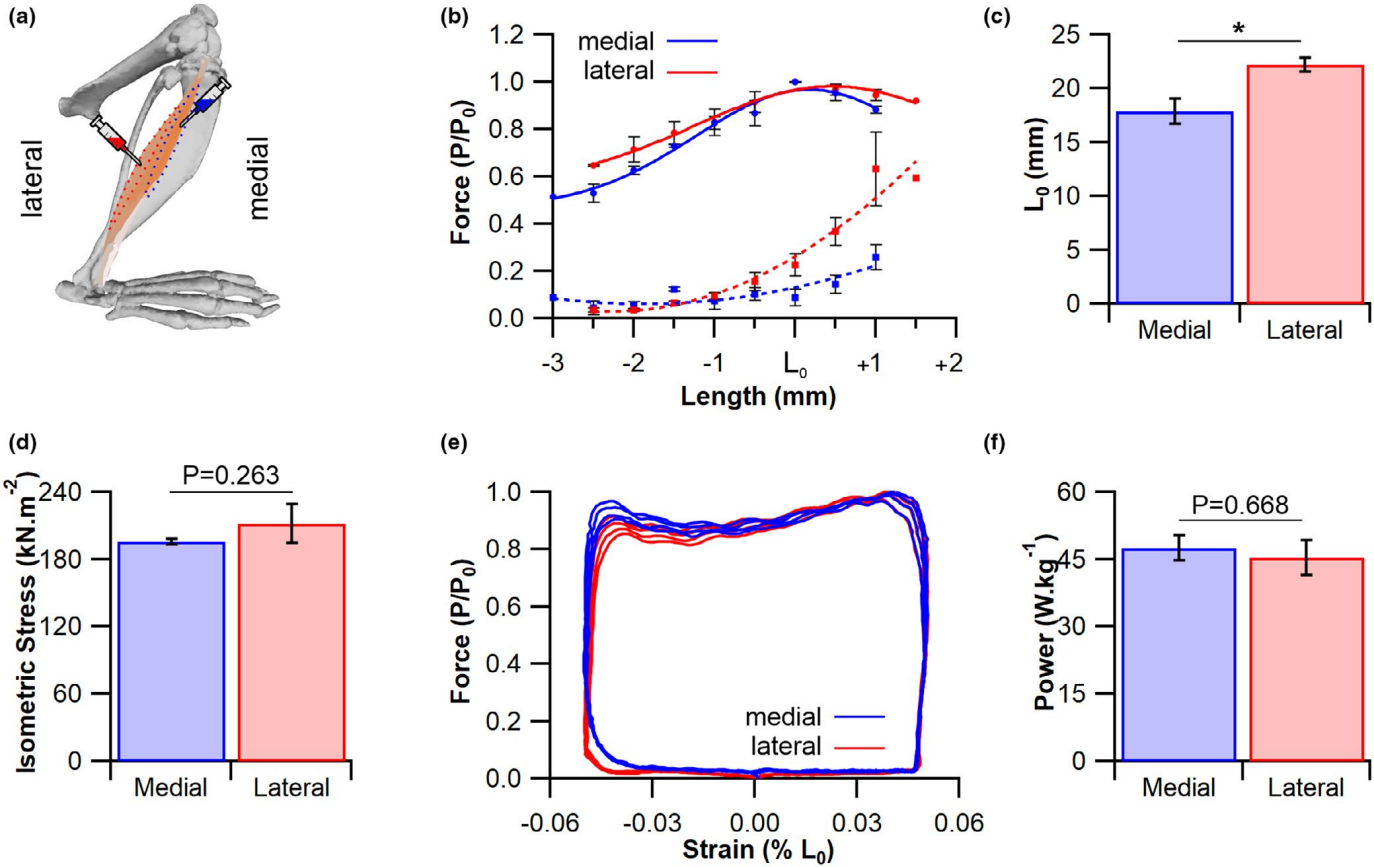
Mechanical properties of the extensor digitorum longus (EDL). (a) The compartmental arrangement of the EDL and sites of retrograde tracer injection. (b) The force–length relationship for the medial (blue) and lateral (red) compartment, with both active (solid lines) and passive (dashed lines) force profiles. (c) Peak length for (d) maximum isometric tetanic stress. (e) An example work loop for the medial (blue) and lateral (red) compartment muscle preparations and (f) the net power generated. Mean ± standard error of the mean (SEM), *p < 0.05. *L*_0_, optimum muscle length to generate force; *P*/*P*_0_, force relative to the peak force during the work loop cycle

Given inherent heterogeneities in oxidative demand (fibre type composition) and oxygen delivery (capillary supply), we sought to explore muscle functional capacity in an ex vivo environment. The active force–length relationship for both compartments of the EDL followed similar profiles, increasing in force approaching *L*_0_, with a defined plateau maintained at +1 mm over *L*_0_ (Figure 3b). However, the passive force properties present with subtly different profiles, with the lateral compartment relative passive force approximately double than that of the medial as length increases beyond *L*_0_ (Figure 3b). Despite similar force–length profiles, the absolute length at which maximal twitch force was generated was significantly different, being lower in the medial compartment compared with the lateral compartment [medial 17.9 ± 1.2 mm vs. lateral 22.3 ± 0.7 mm, *t*(6) = −3.273, *p* = 0.017, Figure 3c]. However, both compartments were able to generate comparable maximal isometric tetanic stress [medial 195.8 ± 2.3 kN m^−2^ vs. lateral 211.9 ± 17.3 kN m^−2^, *t*(6) = −1.234, *p* = 0.263, Figure 3d]. In addition, the muscle work loop technique (Figure 3e) demonstrated that both medial (47.6 ± 2.8 W kg^−1^) and lateral [45.5 ± 3.9 W kg^−1^, *t*(6) = 0.451, *p* = 0.668] muscle compartments were able to generate equivalent net power at a cycle frequency of 7 Hz (Figure 3f).

### 3.4 Heterogeneities within a single motor pool

**FIGURE 4:**
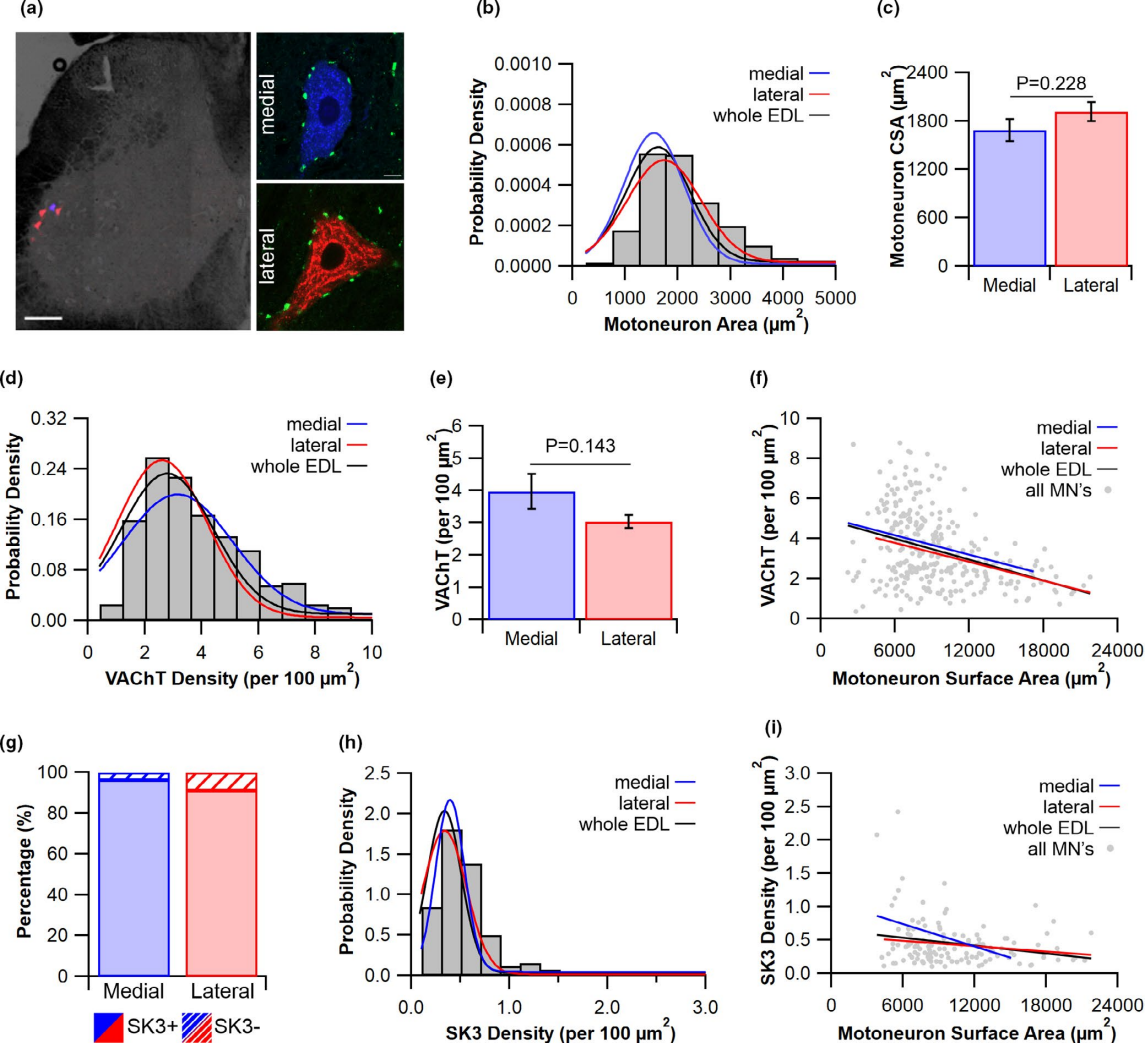
Diversity in morphometrics of a single motor pool. (a) Example low magnification micrograph of spinal cord white matter highlighting fast blue labelled (blue) medial cells and cholera toxin subunit b (CTB) labelled (red) lateral motoneurons. Higher magnification of individual cells co‐stained with vesicular acetylcholine transporter (VAChT, green). (b) Probability density of motoneuron cross‐sectional area (CSA) for the whole extensor digitorum longus (EDL) with Gaussian plots for the whole EDL (black), medial cells (blue) and the lateral cells (red) (b), with (c) the average motoneuron cross‐sectional area. (d) Frequency distribution of VAChT density for the whole EDL with Gaussian plots for the whole EDL (black), medial cells (blue) and the lateral cells (red), with (e) the average density plotted. (f) The significant negative relationship between VAChT and motoneuron surface area across EDL labelled cells. (g) The percentage of positive/negative labelled motoneurons with SK3 in the medial and lateral compartments. (h) Probability density for SK3 density across the entire EDL with Gaussian plots for the whole EDL (black), medial cells (blue) and the lateral cells (red). Finally (i), the lack of any significant relationship between SK3 density and cell surface area. Scale bar = 150 µm. Mean ± standard error of the mean (SEM)

Individual compartments were labelled with retrograde tracers (Figure 4a) in combination with immunohistochemical labelling of SK3 (reportedly expressed only on slow MNs; Deardorff et al., 2021), in an attempt to identify phenotypically distinct MNs within a single motor unit. MN’s were successfully labelled from individual compartments of the EDL (Figure 4a), with medially labelled cells similar in size (1686 ± 134 µm^2^, Figure 4b) to those laterally labelled MNs [1918 ± 121 µm^2^, *t*(10) = −1.283, *p* = 0.226, Figure 4c]. VAChT bouton density on medial MNs (3.97 ± 0.55 100 µm^−2^) was on average 28% higher than those of lateral labelled MNs, though not significantly different [3.04 ± 0.2 100 µm^−2^_,_ *t*(10) = 1.59, *p* = 0.143, Figure 4d,e]. There was a negative relationship between C bouton density and MN surface area (*R* ^2^ = 0.138, *p* < 0.001, Figure 4f).

Our recent work (Kissane et al., 2021) has highlighted that despite the EDL being of a predominantly fast phenotype, with 92% (130 of 141 cells) of all labelled MNs from the motor pool expressing SK3, we were unable to differentially identify phenotypically distinct MNs using such labelling. However, only 4% (1 out of 27 cells) of medial compartment labelled MNs were negative for SK3, while 9% (10 out of 114 cells) of the lateral compartment labelled MNs were negative for SK3 (Figure 4g). The average SK3 density for the medial (0.49 ± 0.09 100 µm^−2^) and lateral [0.38 ± 0.03 100 µm^−2^, *t*(5.105) = 1.277, *p* = 0.287] compartments were not different (Figure 4h); however, the pooled SK3 density was negatively correlated with MN surface area (*R* ^2^ = 0.061, *p* = 0.003, Figure 4i).

## 4 DISCUSSION

**FIGURE 5:**
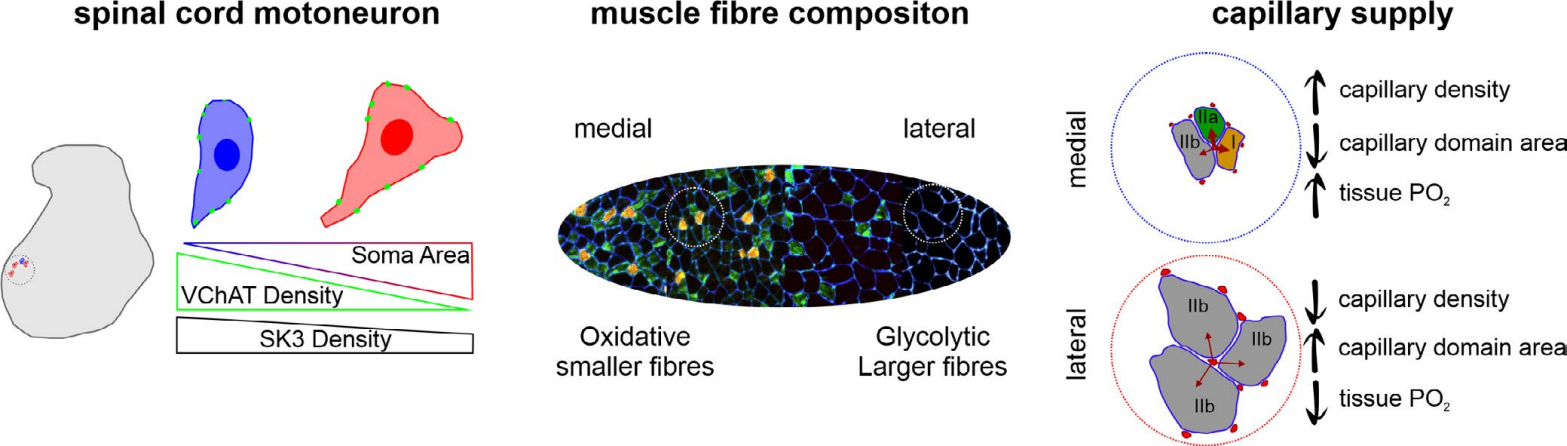
Overview of the extensor digitorum longus (EDL) motor unit heterogeneities. Labelled motoneurons across the EDL motor pool highlight the negative relationship between soma area and VAChT Density and SK3 Density. These motoneurons innervate phenotypically distinct portions of the EDL muscle that is synchronously recruited to provide power for movement. At the level of individual fibres and capillaries heterogeneities in oxygen supply and demand are evident, which when scaled up combine to meet the demands of the muscle, principally maintenance of oxidative phosphorylation in the medial and removal of lactate in the lateral compartment

Through histological assessment of muscle fibre type and capillary supply, mathematical modelling of oxygen transport, assessment of muscle mechanics and retrograde labelling of MNs, our comprehensive overview of the rat EDL motor unit has identified heterogeneity in form and predicted function across multiple organisational levels of a single motor pool (Figure 5). These data emphasise that the motor unit is a tightly regulated system, from the supply of oxygen to individual muscle fibres down to the distribution of ion channels across a MN, and cautions that inadequate sampling techniques (e.g. insufficient random fields of view across histological sample, use of only scale‐dependent indices and inconsistencies in retrograde tracer injections) could lead to false conclusions about motor unit remodelling and plasticity. Understanding these heterogeneities is critical to accurately quantify deficits across the motor pool in response to pathological perturbations, and to aid the development of effective treatment strategies.

### 4.1 Implications of a heterogeneous capillary supply and oxygen demand

The EDL has long been described as a phenotypically fast muscle (Egginton, 1990), and has commonly been used in experimental research as a representative fast, hindlimb muscle (Espino‐Gonzalez et al., 2021; Tickle et al., 2020). However, it may be naïve to class muscles into a single phenotype. Having demonstrated a phenotypic gradient across this mixed muscle and shown regional adaptations to hypoxia (Deveci et al., 2001), we now have refined our approach to identify heterogeneities at the level of an individual capillary and muscle fibre. Our data suggest that within an individual muscle, the oxygen transport system may diverge from assumed symmorphosis (Weibel et al., 1991) where, for example, capillary supply is adequate to meet metabolic demand, and would prioritise maintenance of a relatively constant tissue PO_2_ across the entire muscle. This, however, does not appear to be true of the EDL. With a substantially more dense and tailored vascular supply, the medial compartment appears to prioritise oxygen supply appropriate for tissue oxidative demand, whereas the distinct arrangement of capillaries in the lateral compartment is likely optimised for removal of metabolites (e.g. lactate), a characteristic previously reported between distinctly oxidative and glycolytic muscles (Hudlicka et al., 1987). Future work selectively activating individual compartments through stimulation of individual nerve branches (Balice‐Gordon & Thompson, 1988) in combination with blood gas analysis (Hudlicka et al., 1987) and plasma metabolomics (Hauton et al., 2015) would begin to unpick the role of differential metabolite removal.

### 4.2 Mechanical considerations of heterogenous muscles

Understanding the complexity of muscle fibre heterogeneity on the form and function of a whole muscle is an emerging research area. Recent work has shown fibre length (Bolsterlee et al., 2018; Charles et al., 2019; Kissane et al., 2018) and sarcomere length (Moo et al., 2016) to be heterogeneously distributed throughout the skeletal muscle, and that sarcomeres respond differently across regions in response to whole muscle perturbations (Moo et al., 2016, 2020). Additionally, we have shown that the individual compartments of EDL present distinct fatigue responses during sustained work, with the medial compartment preserving the capacity to maintain force, with impaired relaxation kinetics, whereas the lateral compartment maintains relaxation kinetics with a compromised ability to generate sustained force (Kissane et al., 2018). In contrast, the present data demonstrate that despite considerable phenotypic and morphometric differences between the two compartments, they are able to generate comparable force and net power, under the specified experimental conditions. Unfortunately, to date, there are no published compartmental recordings of *in vivo* activity in the EDL, making it difficult to compare task‐specific functional capacity.

### 4.3 The inherent heterogeneity within an individual motor pool

Few experiments report compartmentally traced MNs (Ishihara et al., 1995), partly due to accessibility challenges at the extreme ends of a muscle, with some researchers opting to crudely divide MN populations based on numerical fibre type composition, e.g. splitting the diaphragm motor pool into tertiles (Rana et al., 2019, 2020). This type of pseudo differentiation of a MN population is challenging, despite 33% of the diaphragm fibres being Type IIb/x fibres (Warren et al., 2020), faster MNs typically innervate more extrafusal fibres than those of slow MNs (Kanning et al., 2010), and therefore exists a great potential to incorrectly classify MNs through the use of equal tertile boundaries.

While histological assessment of phenotypically distinct MNs is problematic we were able to identify MNs from two muscle compartments within the same motor pool. The large proportion of Type IIb/x fibres within both medial and lateral compartments are served by MNs of similar soma area, unlike the difference between compartments seen in the tibialis anterior muscle (Ishihara et al., 1995). However, a trend for smaller MNs in the medial compartment is consistent with the smaller FCSA there. Interestingly, we found a negative relationship between MN size and C‐bouton density, in contrast to that described by Kanning et al. (2010). Therefore, the previous opinion that fast MNs contained higher C‐bouton densities (Hellström et al., 2003) does not hold true for the EDL.

Finally, in light of recent single‐cell transcriptomics of cholinergic neurons, it is perhaps surprising that we were unable to selectively identify slow MNs using SK3 (*KCNN3 gene*) (Kissane et al., 2021), given its supposed enrichment (Blum et al., 2021) and previously reported expression profile (Deardorff et al., 2013, 2014, 2021). While gene expression does not directly translate to protein expression, the discrepancy between the number of positive cells in the current analysis implies that such specificity does not exist. The lack of differential labelling of SK3 within the EDL compartments shows that this small conductance calcium‐activated potassium channel is an inappropriate marker of slow MNs, and reiterates the importance of understanding the heterogeneity of form and function, down to the level of the MN ion channel organisation (Deardorff et al., 2021).

In conclusion, our data demonstrate a significant difference in fibre type composition across the rat EDL, accompanied by a tailored vascular supply to meet metabolic demands. Though quantifiable structural heterogeneities exist, isolated individual compartments were able to produce equivalent power under our experimental conditions. Finally, across the whole motor pool C‐bouton synaptic input correlate negatively with soma size. Together, these data provide a source of reference for the heterogeneities within the rat EDL motor unit and, given the inherent heterogeneities in form and function, emphasise the importance of sampling technique across multiple levels of the motor unit.

## CONFLICT OF INTEREST

All authors confirm that there is no conflict of interest.

## AUTHOR CONTRIBUTIONS

Roger W. P. Kissane conceptualised, designed and undertook the experiments, wrote and edited the manuscript. Samit Chakrabarty provided technical advice and approved the manuscript. Graham N. Askew assisted the isolated muscle experiments, interpret the data and edited the manuscript. Stuart Egginton assisted the design of the study, interpret data and edited the manuscript.

## ETHICAL APPROVAL

All surgical and experimental protocol were approved by the University of Leeds Animal Welfare and Ethics Committee and conducted under the United Kingdom (UK) Animals (Scientific Procedures) Act 1986 (ASPA). This work was conducted following the animal ethics guidelines of the journal.

## Supporting information

Fig S1

## Data Availability

Data may be made available upon request.
