# Supplementary material for: Heterogeneity in form and function of the rat extensor digitorum longus motor unit
Source: J Anat. 2021 Nov 10;240(4):700–10. doi: 10.1111/joa.13590 (PMC8930811; doi:10.1111/joa.13590)
Supplement: Supplementary file 1 — Fig S1 [file JOA-240-700-s001.docx]

# Supplementary Material


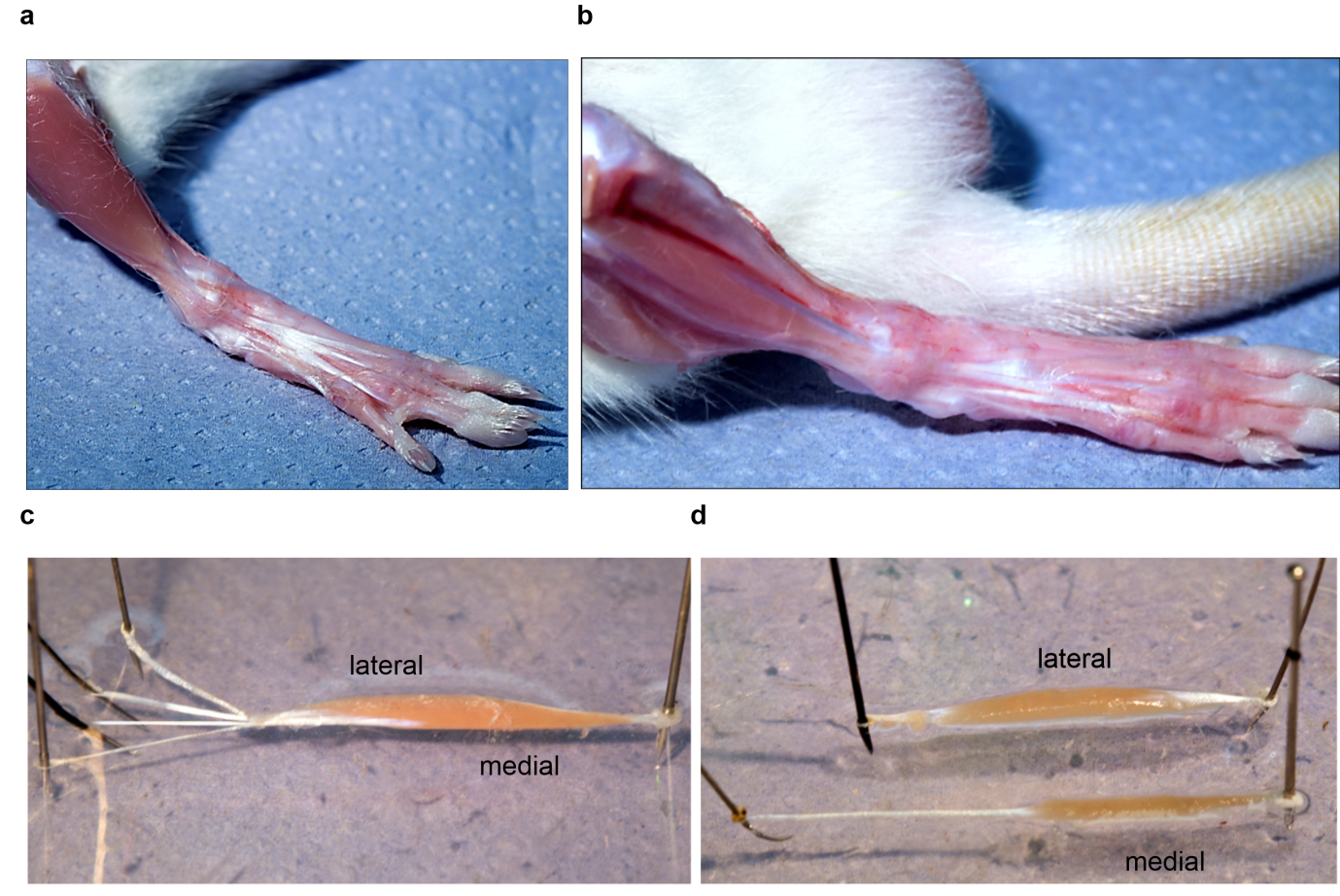


**Supplementary Material 1. The anatomical arrangement of the rat extensor digitorum longus.** (a) Anterior view of the right hind limb of the rat, with the superficial belly of the tibialis anterior (TA) visible, and the multi-tendon arrangement of the extensor digitorum longus (EDL) extending over the tarsals and metatarsal and inserting onto the II-V^th^ phalanges. (b) Removal of the TA reveals the underlying EDL with its extended aponeurosis of the most medial compartment now visible. (c) Pinned out right EDL with each of the distal four tendons anchored. (d) Separated most lateral and medial compartments with the two intermediate compartments removed.
